# The Human Form Divine

**Published:** 1902-10

**Authors:** 


### The Human Form Divine.

The fashion plates of the past half century form a museum of caricature that for grotesqueness no professional artist in that field will ever be able to equal. * * * And yet, semi-sensible women make freaks of themselves by half-way imitations of the idiotic dissections foisted on society by the Parisian man-milliners and the demi-monde. If she rejects it for her own figure, mamma tolerates if she does not even endorse it for her ambitious daughters. Shame on women who have no more womanhood than to surrender to the senseless and distorted fads of the foreign fashion-mongers.—*The Dietetic and Hygienic Gazette.*

